# Proximal-gradient algorithms for fractional programming

**DOI:** 10.1080/02331934.2017.1294592

**Published:** 2017-02-24

**Authors:** Radu Ioan Boţ, Ernö Robert Csetnek

**Affiliations:** ^a^ Faculty of Mathematics, University of Vienna, Vienna, Austria.; ^b^ Faculty of Mathematics and Computer Sciences, Babeş-Bolyai University, Cluj-Napoca, Romania.

**Keywords:** Fractional programming, forward–backward algorithm, convergence rate, convex subdifferential, limiting subdifferential, Kurdyka-ᴌojasiewicz property

## Abstract

In this paper, we propose two proximal-gradient algorithms for fractional programming problems in real Hilbert spaces, where the numerator is a proper, convex and lower semicontinuous function and the denominator is a smooth function, either concave or convex. In the iterative schemes, we perform a proximal step with respect to the nonsmooth numerator and a gradient step with respect to the smooth denominator. The algorithm in case of a concave denominator has the particularity that it generates sequences which approach both the (global) optimal solutions set and the optimal objective value of the underlying fractional programming problem. In case of a convex denominator the numerical scheme approaches the set of critical points of the objective function, provided the latter satisfies the Kurdyka-ᴌojasiewicz property.

## 1. Introduction and preliminaries

Consider the fractional programming problem(1)
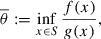



where *S* is a nonempty subset of a real Hilbert space 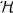, the function *f* is nonnegative and the function *g* is positive on *S*. One of the classical methods to handle (1) is Dinkelbach’s procedure (see [1,2]) which relates it to the following optimization problem(2)
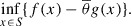



If (1) has an optimal solution 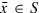, then this is also an optimal solution to (2) and the optimal objective value of the latter is equal to zero. Vice-versa, if (2) has 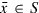 as an optimal solution and its optimal objective value is equal to zero, then 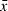 is an optimal solution to (1), too. This shows that finding an optimal solution to (1) can be approached by algorithms which solve (2). However, one drawback of this procedure is that this can be done in the very restrictive case when the optimal objective value of (1) is known.

One can find in the literature (see [1–5]) an iterative scheme which, in the attempt to overcome this drawback in finite-dimensional spaces, requires the solving in each iteration 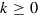 of the optimization problem(3)
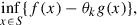



while 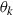 is updated by 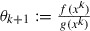, where 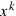 is an optimal solution of (3). However, solving in each iteration an optimization problem of type (3) can be as expensive and difficult as solving the fractional programming problem (1).

The aim of this note is to propose an alternative to this approach. Namely, we formulate two iterative schemes for solving (1), where 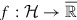 is proper, convex and lower semicontinuous and 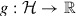 is differentiable with Lipschitz continuous gradient and either concave or convex. Instead of solving in each iteration (3), the proposed iterative methods perform a gradient step with respect to *g* and a proximal step with respect to *f*. In this way, the functions *f* and *g* are processed separately in each iteration. A further advantage of the algorithm investigated in case *g* is concave comes from the fact that it generates sequences that concomitantly approach the set of optimal solutions and the optimal objective value of (1). The second numerical scheme, proposed in case *g* is convex, has the particularity that it approaches the set of critical points of the objective function of (1), provided the latter satisfies the Kurdyka-ᴌojasiewicz property.

For the notations used in this paper we refer the reader to [6–9]. Let 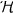 be a real Hilbert space with *inner product* 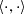 and associated *norm* 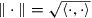. The symbols 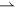 and 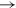 denote weak and strong convergence, respectively.

For a function 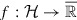, where 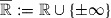 is the extended real line, we denote by 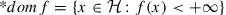 its *effective domain* and say that *f* is *proper* if 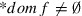 and 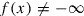 for all 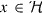. The *subdifferential* of *f* at 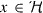, with 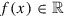, is the set 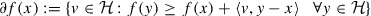. We take by convention 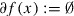, if 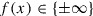. Let 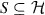 be a nonempty set. The *indicator function* of *S*, 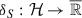, is the function which takes the value 0 on *S* and 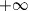 otherwise.

An efficient tool for proving weak convergence of a sequence in Hilbert spaces (without a priori knowledge of its limit) is the Opial Lemma, which we recall in the following.

Lemma 1: [Opial] Let *C* be a nonempty set of 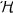 and 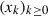 be a sequence in 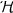 such that the following two conditions hold:

(a) for every 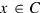, 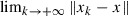 exists;
(b) every weak sequential cluster point of 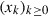 is in *C*;

Then 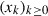 converges weakly to an element in *C*.

When proving the first part of the Opial Lemma, one usually tries to show that for every 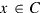 the sequence 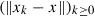 fulfils a Fejér-type inequality. In this sense, the following result is very useful.

Lemma 2: Let 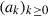, 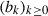 and 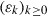 be real sequences. Assume that 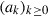 is bounded from below, 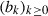 is nonnegative, 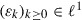 and 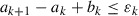 for every 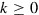. Then 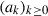 is convergent and 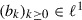.

The following summability result will be useful in Section 2.2.

Lemma 3: Let 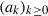 and 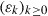 be nonnegative real sequences, such that 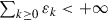 and 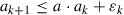 for every 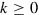, where 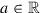, 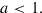 Then 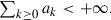

Finally, the descent lemma which we recall next is a helpful tool in the convergence analysis of the algorithms proposed in this manuscript.

Lemma 4: [see [10, Lemma 1.2.3]] Let 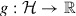 be (Fréchet) differentiable with *L*-Lipschitz continuous gradient. Then
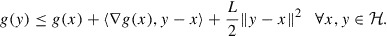

## 2. Two proximal-gradient algorithms

In this section, we propose two proximal-gradient algorithms for solving (1) and investigate their convergence properties. We treat the situations when *g* is either a convex or a concave function separately.

### 2.1. Concave denominator

The problem that we investigate throughout this subsection has the following formulation.

Problem 5: We are interested in solving the fractional programming problem(4)
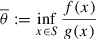

where 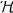 is a real Hilbert space, *S* is a nonempty, convex and closed subset of 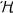, 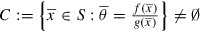 and the following conditions hold:
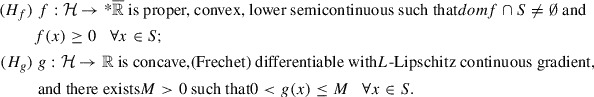

To this aim we propose the following algorithm.


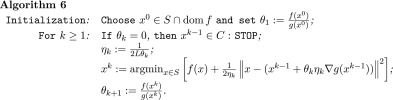


We are now in position to present the convergence statement of this algorithm. To this end, we assume that the algorithm does not stop after finitely many iterations.

Theorem 6: In the setting of Problem 5, consider the sequences generated by Algorithm . The following statements hold:

(i) The sequence 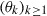 is nonincreasing and 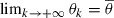. Moreover,(5)
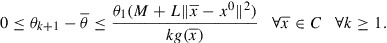

(ii) Additionally, assume that 
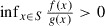
. Then the sequence 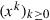 converges weakly to an element in *C*.

According to the first-order optimality conditions, we have(6)
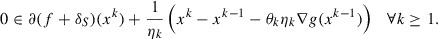



A direct consequence of the definition of the convex subdifferential is the inequality(7)
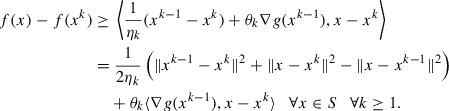



Invoking the concavity of *g* and using that 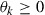, we have(8)
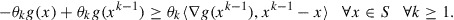



Combining (7) and (8), we obtain(9)
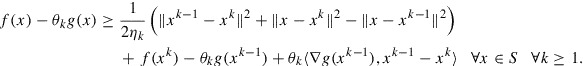



Lemma 4 applied to the function 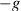 yields the inequality
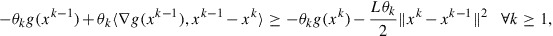



hence from (9) we derive
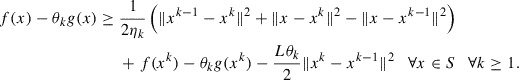



Taking into account the relation 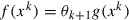 and the way 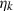 is defined, we obtain for every 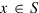 and 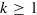 the inequality(10)
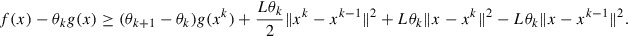

(i) Taking 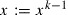 in (10) we get(11)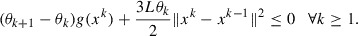 This further implies that 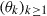 is a nonincreasing sequence, hence convergent, since it is bounded from below by 0. Consider now an arbitrary 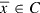 and take 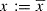 in (10). We derive(12)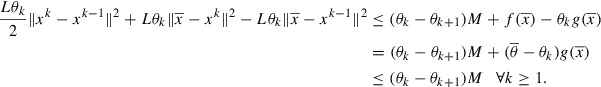 This yields the inequality(13)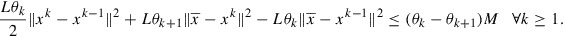 Since 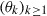 is bounded from below by 0, the sequence on the right-hand side of inequality (13) belongs to 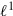. We derive from Lemma 2 that(14)
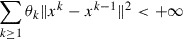
 and(15)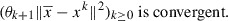 Coming back to (12) and using 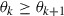, we obtain(16)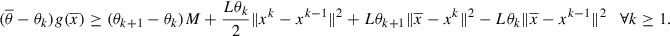 Relying on (14) and (15) and the convergence of the sequence 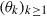, the right-hand side of the above inequality is a sequence which converges to 0 as 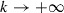. Invoking also the fact that 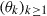 is bounded from below by 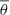, we conclude that 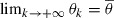. Let us prove now the convergence rate result stated in (5). Let 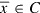 and 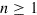 be arbitrary. From (11) we obtain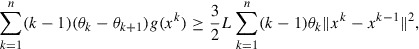 hence
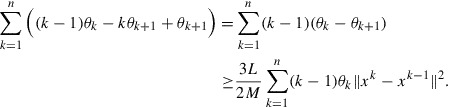
 Noticing the telescoping sum in the left-hand side of the previous inequality, we obtain(17)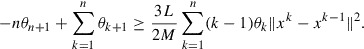 Summing up the inequalities in (16) for *k* from 1 to 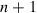 we obtain
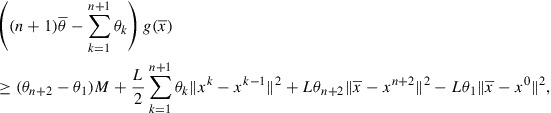
 hence(18)
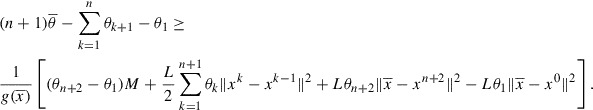
 Summing up the inequalities (17) and (18) and discarding the nonnegative terms on the right-hand side we derive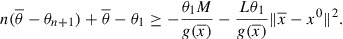 Noticing that 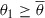, the last inequality implies (5) after rearranging the terms.
(ii) For the remaining of the proof we assume that 
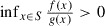
. In this situation, 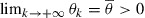 and from (14) and (15) we derive(19)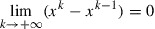 and(20)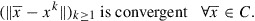 Thus the first condition in the Opial Lemma is fulfilled. Consider now a subsequence 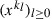 of 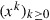 that weakly converges to 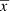 as 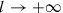. From (6) we deduce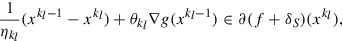 hence(21)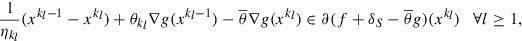 due to the concavity of *g* and 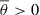. Since for every 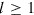 we have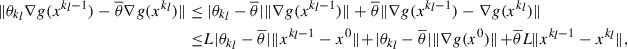 from (i), (19) and the fact that 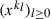 is bounded, we conclude that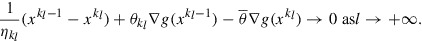 Since 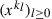 weakly converges to 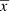 as 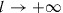, from (21) and the fact that the graph of the convex subdifferential of a proper, convex and lower semicontinuous function is sequentially closed with respect to the weak-norm topology (see [7, Proposition 20.33]), we derive that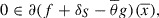 hence 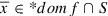. The definition of the convex subdifferential yields the inequality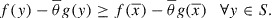 From here, by choosing 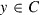, we get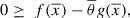 hence(22)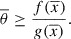 Relation (22) implies now that 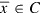. Thus the second condition in the Opial Lemma is also fulfilled. The conclusion follows now from Lemma 1.

### 2.2. Convex denominator

In this subsection we consider the case when *g* is a convex function.

Problem 7: We are interested in solving the fractional programming problem(23)
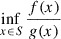

where 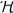 is a real Hilbert space, *S* is a nonempty, convex and closed subset of 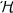, and the following conditions hold:
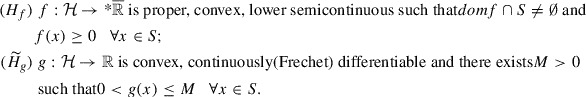

The algorithm we propose in this context has the following formulation.


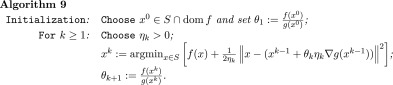


The proof of the first result in this subsection reveals the fact that when *g* is convex one cannot expect convergence of the whole sequence 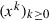. Furthermore, if this is the case, then the limit is not necessarily an optimal solution of (23), but a critical point of the objective function 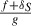 in the sense of the limiting subdifferential. In order to explain this notion, we need some prerequisites of nonsmooth analysis.

For the following generalized subdifferential notions and their basic properties, we refer to [11,12]. Let 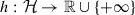 be a proper and lower semicontinuous function. If 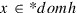, we consider the *Fréchet (viscosity) subdifferential* of *h* at *x* as being the set
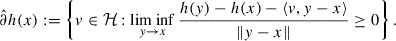



For 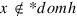 we set 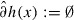. The *limiting (Mordukhovich) subdifferential* is defined at 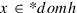 by
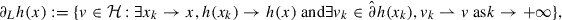



while for 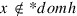, one takes 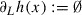. Therefore 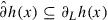 for each 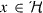.

When *h* is continuously differentiable around 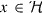 we have 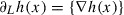. Notice that in case *h* is convex, these two subdifferential notions coincide with the *convex subdifferential*, thus 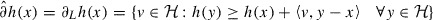 for all 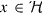.

The Fermat rule reads in this nonsmooth setting: if 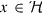 is a local minimizer of *h*, then 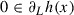. An element 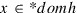 fulfilling this inclusion relation is called *critical point* of the function *h*. The set of all critical points of *h* is denoted by 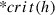.

The convergence of Algorithm () is stated in the following theorem.

Theorem 8: In the setting of Problem 7, consider the sequences generated by Algorithm such that the additional condition 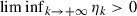 is satisfied. The following statements hold:

(i) The sequence 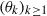 is nonincreasing, hence convergent. Moreover,
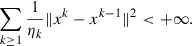

(ii) For every (strong) limit point 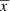 of 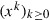, it holds 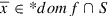 and 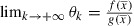. If we additionally have that 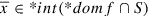, then 
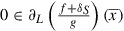
.

As already seen in the proof of Theorem 6, we have(24)
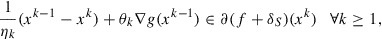



and(25)
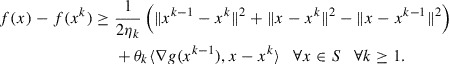



By choosing 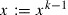 in (25) we obtain
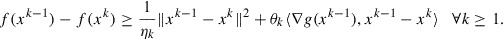



Further, by combining this with
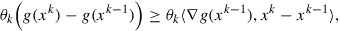



we obtain(26)
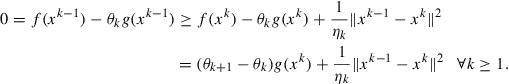

(i) From (26) we obtain that 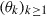 is nonincreasing, hence convergent, since it is bounded from below by 0. Moreover, from (26) we obtain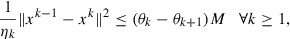 hence 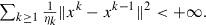

(ii) Without losing the generality, we assume that 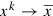 as 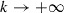. Since *S* is closed, we have 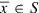. By choosing 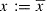 in (25), we obtain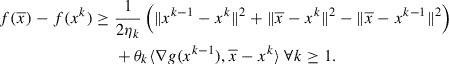 By using (i), one can see that the right-hand side of the above inequality converges to 0 as 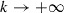. Hence, 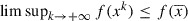. Since *f* is lower semicontinuous, the reverse inequality is also true, thus
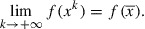
 Furthermore, due to the continuity of *g*, we have
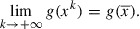
 Let us denote by 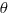 the limit of the sequence 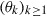. Passing to the limit as 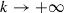 in the relation which defines 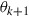 in Algorithm , we obtain(27)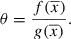 By using again the closedness property of the graph of the convex subdifferential, from (24) and (i) we obtain(28)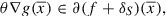 hence 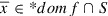. Assume now that 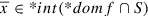. In this situation 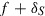 is Lipschitz continuous around 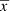 (see [7, Theorem 8.29]). From (27) and (28) we obtain
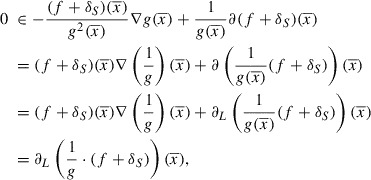
 where the last equality makes use of [11, Corollary 1.111(i)].

Remark 9:

(a) The main ingredient in the proof of the second statement of the above theorem is the rule for the limiting subdifferential of the product (or quotient) of locally Lipschitz continuous functions. We notice that similar rules are valid also for the Clarke subdifferential (see [13, Exercise 10.21]).
(b) Whenever 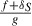 is a convex function, we obtain in the hypotheses of the above theorem that 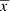 is a global optimal solution of (23) and 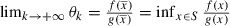.

In the remaining of this subsection, we address the question whether one can guarantee the convergence of the whole sequence 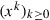 generated in Algorithm . We will see that this is ensured whenever the objective function of (23) satisfies the *Kurdyka-ᴌojasiewicz property*. To this end we recall some notations and definitions related to the latter.

For the remaining of this section, we suppose that 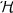 is finite dimensional. For 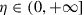, we denote by 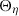 the class of concave and continuous functions 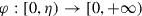 such that 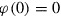, 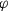 is continuously differentiable on 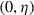, continuous at 0 and 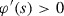 for all 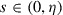. In the following definition (see [14,15]) we use also the *distance function* to a set, defined for 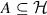 as 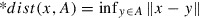 for all 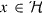.

Definition 10: [Kurdyka-ᴌojasiewicz property] Let 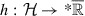 be a proper and lower semicontinuous function. We say that *h* satisfies the *Kurdyka-ᴌojasiewicz (KL) property* at 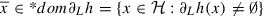 if there exists 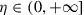, a neighbourhood *U* of 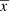 and a function 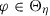 such that for all *x* in the intersection
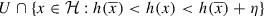

the following inequality holds
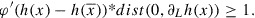

If *h* satisfies the KL property at each point in 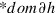, then *h* is called a *KL function*.

The origins of this notion go back to the pioneering work of ᴌojasiewicz [16], where it is proved that for a real-analytic function 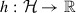 and a critical point 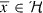 (that is 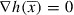), there exists 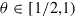 such that the function 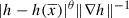 is bounded around 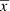. This corresponds to the situation when 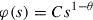, where 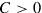. The result of ᴌojasiewicz allows the interpretation of the KL property as a re-parametrization of the function values in order to avoid flatness around the critical points. Kurdyka [17] extended this property to differentiable functions definable in an o-minimal structure. Further extensions to the nonsmooth setting can be found in [14,18–20].

One of the remarkable properties of KL functions is their ubiquity in applications, according to [15]. To this class of functions belong semi-algebraic, real sub-analytic, semiconvex, uniformly convex and convex functions satisfying a growth condition. We refer the reader to [14,15,18,22] and the references therein for more details regarding KL functions and illustrating examples.

An important role in our convergence analysis will be played by the following uniformized KL property given in [15, Lemma 6].

Lemma 11: Let 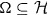 be a compact and connected set and let 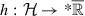 be a proper and lower semicontinuous function. Assume that *h* is constant on 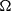 and *h* satisfies the KL property at each point of 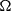. Then there exist 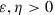 and 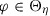 such that for all 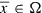 and for all *x* in the intersection(29)
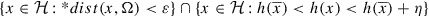

the following inequality holds(30)
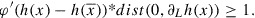

The techniques used below are well-known in the community dealing with algorithms for optimization problems involving functions with the Kurdyka-ᴌojasiewicz property (see [15,21,23,24]). We show that this approach can be used also for fractional programming problems.

In the following, we denote by 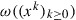 the set of cluster points of the sequence 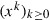. The first statement in the next result is a direct consequence of Theorem 8, while the other statements can be proved similar to [15, Lemma 5], where it is noticed that (b) and (c) are generic for sequences satisfying the relation 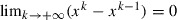.

Lemma 12: In the setting of Problem 7, let 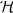 be finite dimensional and consider the sequences generated by Algorithm such that the additional condition
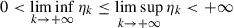

is satisfied. Assume that 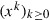 is bounded. The following statements hold:

(a)

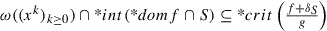
;
(b)

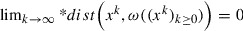
;
(c)

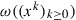
 is nonempty, compact and connected;
(d)

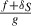
 is finite and constant on 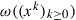.

Remark 13: Suppose that 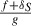 is coercive, that is
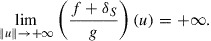

Then the sequence 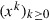 generated by Algorithm is bounded. Indeed, this follows from the fact that 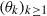 is nonincreasing and the lower level sets of 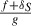 are bounded.

We give now the main result concerning the convergence of the whole sequence 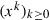.

Theorem 14: In the setting of Problem 7, let 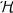 be finite dimensional, 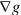 be *L*-Lipschitz continuous, and consider the sequences generated by Algorithm under the additional conditions 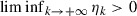, 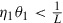 and 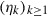 nonincreasing. Assume that 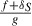 is a KL function. Moreover, suppose that 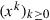 is bounded and there exists 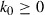 such that 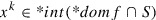 for all 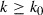. Then the following statements are true:

(a)

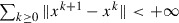
;
(b) there exists 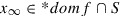 such that 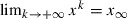. If additionally 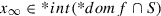, then 
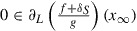
.

(a) Consider the sequences generated by Algorithm . According to Lemma 12 we can choose an element 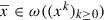. By Theorem 8(ii), we have 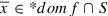 and 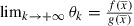. We separately treat the following two cases.
  (I) There exists 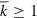 such that 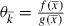. Since 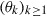 is nonincreasing, we have 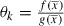 for every 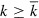. By using (26), we deduce that the sequence 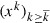 is constant. From here the conclusion follows automatically.
  (II) For all 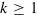 it holds 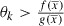. Take 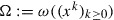.
  In virtue of Lemma 12(c) and (d) and Lemma 11, the KL property of 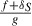 leads to the existence of positive numbers 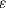 and 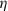 and a concave function 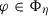 such that for all(31)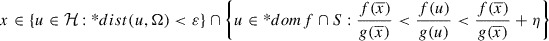 one has(32)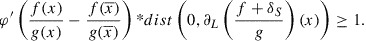 Let 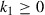 be such that 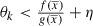 for all 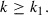 According to Lemma 12(b), there exists 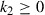 such that 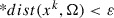 for all 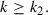 Hence the sequence 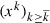, where 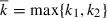, belongs to the intersection (31). So we have (see (32))(33)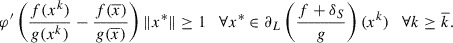 Since 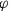 is concave, it holds for all 
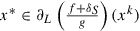
 and for all 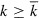

(34)
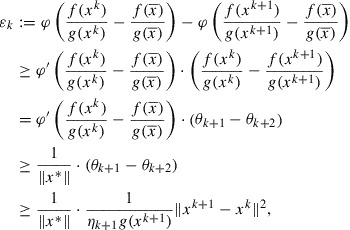
 where the last inequality follows from (26). Further, by using (24) and [11, Corollary 1.111(i)], we have that for every 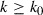


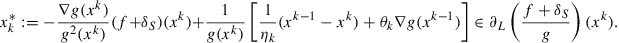
 Furthermore, notice that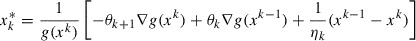 and relying on the Lipschitz continuity of the gradient we derive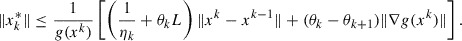 Altogether, from (35) we obtain for every 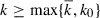


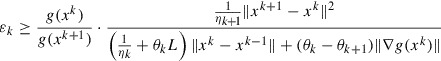
 and from here(35)
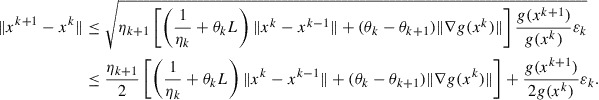
 Further, we observe that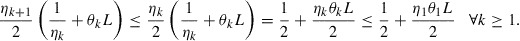 Moreover, 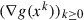 is bounded and 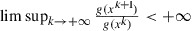, due to 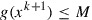 (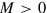) and 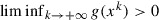, which follows from the continuity of *g*, the fact that 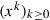 is bounded and Theorem 8(ii). Thus there exist some positive constants 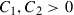 and 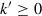 such that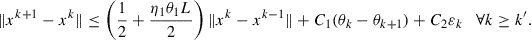 The conclusion follows from Lemma 3 by noticing that 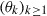 and 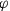 are bounded from below.
(b) It follows from (a) that 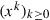 is a Cauchy sequence, hence it is convergent. The conclusion follows from Theorem 8.

 

## 3. Future work

We point out some open questions to be followed in the future related to the solving of the fractional programming problem under investigation:

(1) Is it possible to evaluate in each iteration the functions *f* and 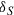 separately, which would actually mean that the set *S* is addressed in the algorithm by means of its projection operator?
(2) How to incorporate in Algorithm some extrapolation terms in the sense of Nesterov in order to improve its speed of convergence?
(3) Can one consider also other situations, for instance when *f* is smooth and *g* is nonsmooth, or even the more general case where both functions are nonsmooth?

 
